# Gastric cancer treatment in Brazil: a multicenter study of the Brazilian Gastric Cancer Association

**DOI:** 10.1590/0100-6991e-20253815_en

**Published:** 2025-04-28

**Authors:** Marcus Fernando Kodama Pertille Ramos, Marina Alessandra Pereira, Thiago Francischetto Ribeiro, Oddone Braghiroli, Felipe José Fernandez Coimbra, Marco Antônio Gonçalves Rodrigues, Flavio Duarte Sabino, Ulysses Ribeiro, Ronaldo Mafia Cuenca, Felipe Carvalho Victer, Flávio Daniel Saavedra Tomasich, Geraldo Ishak, Antonio Nocchi Kalil, Álvaro Antônio Bandeira Ferraz, Luis Fernando Moreira, Claudemiro Quireze, Nelson Adami Andreollo, Osvaldo Antônio Prado Castro, Fernando Antônio Siqueira Pinheiro, Antônio Carlos Weston

**Affiliations:** 1 - Hospital das Clinicas HCFMUSP, Faculdade de Medicina, Universidade de São Paulo, Instituto do Câncer - São Paulo - SP - Brasil; 2 - Hospital Aristides Maltez, Liga Bahiana Contra o Câncer, Cirurgia - Salvador - BA - Brasil; 3 - Hospital Universitário Professor Edgard Santos, Universidade Federal da Bahia, Departamento de Anestesia e Cirurgia - Salvador - BA - Brasil; 4 - Hospital AC Camargo Cancer Center, Departamento de Cirurgia Abdominal - São Paulo - SP - Brasil; 5 - Hospital das Clinicas da Universidade Federal de Minas Gerais, Departamento de Cirurgia - Belo Horizonte - MG - Brasil; 6 - Instituto Nacional do Câncer, Serviço de Cirurgia Abdômino-pélvica - Rio de Janeiro - RJ - Brasil; 7 - Hospital Universitário de Brasília, Universidade de Brasília, Departamento de Cirurgia - Brasilia - DF - Brasil; 8 - Hospital Universitário Clementino Fraga Filho, Universidade Federal do Rio de Janeiro, Serviço de Cirurgia Geral - Rio de Janeiro - RJ - Brasil; 9 - Hospital Erasto Gaertner, Departamento de Clínica Cirúrgica - Curitiba - PR - Brasil; 10 - Hospital Universitário João de Barros Barreto, Universidade Federal do Pará, Serviço de Cirurgia Geral e Aparelho Digestivo - Belém - PA - Brasil; 11 - Santa Casa de Misericórdia de Porto Alegre, Serviço de Cirurgia Geral e Cancerologia Cirúrgica - Porto Alegre - RS - Brasil; 12 - Hospital das Clinicas, Universidade Federal de Pernambuco, Serviço de Cirurgia Geral - Recife - PE - Brasil; 13 - Hospital de Clinicas de Porto Alegre, Serviço de Cirurgia Geral - Porto Alegre - RS - Brasil; 14 - Hospital das Clinicas da Universidade Federal de Goiás, Unidade de Clínica Cirúrgica - Goiânia - GO - Brasil; 15 - Hospital de Clinicas da Universidade Estadual de Campinas, Departamento de Cirurgia - Campinas - SP - Brasil; 16 - Santa Casa de Misericórdia de São Paulo, Departamento de Cirurgia - São Paulo - SP - Brasil; 17 - Hospital Universitário Walter Cantídio, Universidade Federal do Ceará, Departamento de Cirurgia - Fortaleza - CE - Brasil; 18 - Associação Brasileira de Câncer Gástrico - São Paulo - SP - Brasil

**Keywords:** Gastric Neoplasms, Gastrectomy, Survival Analysis, Neoplasias Gástricas, Gastrectomia, Análise de Sobrevivência

## Abstract

**Introduction:**

Gastric cancer (GC) has distinct characteristics and management according to the region of the world, and the objective of our study was to evaluate how it is being managed in Brazil.

**Methods:**

This is a multicenter study that involved 18 oncology referral centers. Data were collected using the REDCap platform and compiled at the end of one year.

**Results:**

All Brazilian regions were represented, and 635 patients were included. Most patients were from the Southeast (40.6%) and Northeast (29.6%) regions. The mean age was 62 years, with a predominance of males. Most patients (84.6%) had good performance status, with an ECOG score of 1-2. Less than 10% of patients were covered by medical insurance. A quarter of the patients underwent diagnostic laparoscopy, but endoscopic ultrasound and PET scans were rarely performed. The cT3 category was the most common (40.6%), lymph node involvement was described in 48.9%, and distant metastases, in 14.4% of the staging exams. The final cTNM staging was III (29.4%), II (26%), I (24.2%) and IV (20.5%). Most patients underwent surgery with curative intent (74.4%) and open access (82.8%). Preoperative chemotherapy was performed in 37.2% of cases, and the most common surgical procedures were subtotal gastrectomy (45.3%) and total gastrectomy (33.1%).

**Conclusion:**

The present study allowed us to evaluate the current panorama of surgical treatment of Gastric Cancer, representing all regions of Brazil. Stage III, distal, and diffuse tumors continue to be prevalent in Brazil, and there has been relevant use of diagnostic laparoscopy, preoperative chemotherapy, and minimally invasive surgery.

## RESUMO

**Introdução:**

O câncer gástrico (CG) apresenta características e manejos diferentes de acordo com a região do mundo e o objetivo do nosso estudo foi avaliar como ele está sendo realizado no Brasil.

**Métodos:**

Este é um estudo multicêntrico que envolveu 18 centros de referência oncológicos. Os dados foram coletados usando a plataforma REDCAP e compilados ao final de 1 ano.

**Resultados:**

Todas as regiões brasileiras foram representadas e 635 pacientes foram incluídos. A maioria dos pacientes era das regiões Sudeste (40,6%) e Nordeste (29,6%). A média de idade foi de 62 anos, com predomínio do sexo masculino. A maioria dos pacientes (84,6%) tinha bom desempenho clínico, com ECOG de 1-2. Menos de 10% dos pacientes eram cobertos por seguro médico. Um quarto dos pacientes foi submetido a laparoscopia diagnóstica, mas ultrassonografia endoscópica e PET scans raramente foram realizados. A categoria cT3 foi a mais comum (40,6%), envolvimento de linfonodos foi descrito em 48,9% e metástases à distância em 14,4% dos exames de estadiamento. O estadiamento cTNM final foi III (29,4%), II (26%), I (24,2%) e IV (20,5%). A maioria dos pacientes foi submetida à cirurgia com intenção curativa (74,4%) e via de acesso aberta (82,8%). Quimioterapia pré-operatória foi realizada em 37,2% dos casos e os procedimentos cirúrgicos mais comuns foram gastrectomia subtotal (45,3%) e gastrectomia total (33,1%).

**Conclusão:**

O presente estudo permitiu avaliar o panorama atual do tratamento cirúrgico do CG, representando todas as regiões do Brasil. Tumores com estádio III, distais e difusos continuam predominantes no Brasil e foi observado um uso relevante da laparoscopia diagnóstica, quimioterapia pré-operatória e cirurgia minimamente invasiva.

## INTRODUCTION

Gastric cancer (GC) remains a major global health problem, with more than one million cases diagnosed annually. In Brazil, it is estimated that there were 21,480 new cases of gastric cancer in 2023, being the fifth most incident tumor^1^.

The main therapeutic modality for locally advanced GC continues to be surgical resection with negative margins and associated lymphadenectomy^2^ ^,^ ^3^. In recent years, multimodal treatment including chemotherapy (CTX), radiation therapy (RT), and surgery have improved patient survival. This strategy has also made it possible to perform conversion surgery with curative intent in cases previously considered to be without therapeutic possibilities^4^ ^,^ ^5^. Advances in the indication of endoscopic resection procedures and minimally invasive surgery have also been incorporated into the therapeutic arsenal. This increase in therapeutic options has made it necessary for professionals who treat GC to have greater expertise in the disease^6^.

In this scenario, the Brazilian Association of Gastric Cancer (ABCG) was founded on June 14, 1999, and brings together specialists in GC treatment from all over the country. It is a multidisciplinary organization and includes clinical oncologists, surgeons, radiologists, pathologists, endoscopists, clinicians, and epidemiologists. ABCG conducts teaching activities, such as the Brazilian Gastric Cancer Journey, and supports various events related to the treatment of GC. ABCG also participates in the International Gastric Cancer Congress (IGCC), which takes place every two years in countries around the world. The IGCC is organized by the International Gastric Cancer Association (IGCA), and ABCG is its representative in Brazil.

Brazil is a country of continental dimension, with many regional peculiarities and disparities that directly impact the diagnosis, treatment, and prognosis of patients with GC^7^. Thus, evaluating the current management of the GC, including all regions of the country, is within the scope of activities performed by the ABCG. Thus, the present study aims to assess the current state of GC treatment in Brazil.

## METHODS

This is a multicenter study that involved 18 national centers from June 2022 to June 2023. All the centers invited to participate have known experience in GC treatment, were linked to Universities or Oncology High Complexity Care Centers/Units (CACON and UNCACON) and have representatives who participate in ABCG activities. We included patients with a diagnosis of gastric adenocarcinoma confirmed by biopsy who underwent any surgical procedure related to GC treatment. We excluded patients with histological types other than adenocarcinoma (GIST, neuroendocrine, lymphoma) and the ones undergoing surgical procedures associated with the treatment of complications of metastatic disease.

The surgical procedures and technique followed the recommendations of the Japanese Gastric Cancer Association (JGCA) and the II Brazilian Consensus on Gastric Cancer, organized by ABCG^2^ ^,^ ^3^. To collect the study data, we developed a database using the Research Electronic Data Capture (REDCap) web platform. Project participants collected data at each center. The data were kept independently at each center and were compiled only at the end of the first year. Clinical variables included age, sex, body mass index (BMI), American Society of Anesthesiologists (ASA) physical status, Eastern Cooperative Oncology Group (ECOG) performance status, hemoglobin, and serum albumin levels. Tumor characteristics included size, location, Lauren’s histological type, differentiation, and clinical staging (cTNM). We also recorded the use of diagnostic laparoscopy, positron emission tomography (PET), and endoscopic ultrasound (EUS). In addition, we evaluated data on the use of chemotherapy (CTX) and the type of surgical procedure.

The study was approved by the local ethics committee of all centers and registered online at Plataforma Brasil (CAAE: 41844820.5.1001.0068). The Hospital das Clínicas of the University of Sao Paulo Medical School (CCEP 1833/20) was the main investigative center. The patients’ informed consent was waived and there was no funding to conduct the study.

Data were expressed as mean (with standard deviation, SD±) for continuous variables and as numbers with frequency (in percentage) for categorical data. All percentages were calculated to reach the value of 100% according to the available data for each variable (missing data excluded). Statistical analyses were performed using SPSS software, version 20 (SPSS, Chicago, IL).

## RESULTS

**Figure 1:**
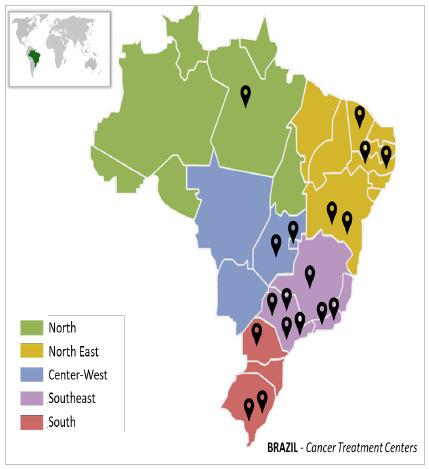
Geographical distribution in Brazil of all centers participating in the study.

For one year, 635 patients were included by the participating centers. All Brazilian regions were covered by at least one center participating in the study (Figure 1).

**Figure 2:**
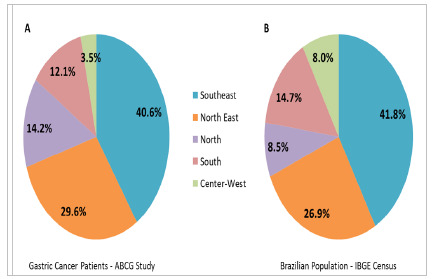
Frequency of patients included in the study by region of Brazil (A); and distribution of the Brazilian population according to the IBGE Census, by region (B).

Figure 2 shows the distribution of the cases according to each region of the country. Most patients were from the Southeast, representing 40.6% of the cases, followed by the Northeast region (29.6%).

Table 1 summarizes the patients’ characteristics. The mean age was 62 years, with a predominance of males. Most patients (84.6%) had good clinical condition, with ECOG performance status 1 and 2. Among the centers included, only two had public and private health care patients (one in the Southeast region and one in the South). Of these, 58.7% of cases corresponded to private care (45/75 cases). Considering all the cases included (n: 635), less than 10% of the patients were covered by health insurance.

**Table 1:** Clinical characteristics of all patients included.

Variable		n	%
Age			
	Mean (SD)	62 (12.4)	
Sex			
	Female	260	41,7
	Male	363	58,3
BMI (Kg/m²)			
	Mean (SD)	24.1 (5.3)	
ASA			
	I	74	15,8
	II	276	58,8
	III	115	24,5
	IV	4	0,9
ECOG			
	0	121	26,6
	1	264	58,0
	2	61	13,4
	3	9	2,0
Hemoglobin (g/dL)			
	Mean (SD	12 (2.4)	
	≤11	150	32.2
Albumin (g/dL)			
	Mean (SD	3.8 (0.7)	
	≤3,5	62	29.2
Type of Hospital			
	Private	44	8,1
	Public (SUS)	500	91,9

SD: standard deviation; BMI: body mass index; ASA: American Society of Anesthesiologists; ECOG: Eastern Cooperative Oncology Group.

For clinical staging, 153 patients underwent diagnostic laparoscopy. Of these, 47 (30.7%) had metastases. Endoscopic ultrasonography and PET scans were rarely performed (Table 2). 

**Table 2:** Clinical staging and pathological characteristics of all patients included.

Variables	n	%
Diagnostic laparoscopy		
Not performed	466	75,3
Yes - P0	106	17,1
Yes - P1	47	7,6
Endoscopic ultrasound		
No	598	96,6
Yes	21	3,4
PET scan		
No	604	97,6
Yes	15	2,4
Tumor location		
Lower third	296	47,1
Middle third	223	35,5
Upper third	99	15,8
Whole organ	10	1,6
Tumor size (cm)		
Mean (SD)	4,9 (3,1)	
Lauren’s histological type		
Intestinal / indeterminate	217	34,2
Diffuse/Mixed	334	52,6
Histological differentiation		
Bem/moderada	191	33,6
Poorly	377	66,4
cTNM clinical staging		
I	144	24,2
IIA	33	5,5
IIB	122	20,5
III	175	29,4
IVA	34	5,7
IVB	88	14,8

SD: standard deviation; PET: positron emission tomography.

Almost half of the tumors were in the distal portion of the stomach, and Lauren’s diffuse histological type was the most common (60.6%).

**Figure 3:**
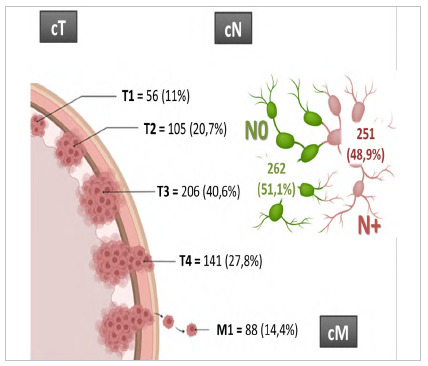
TNM clinical staging. Frequency of cT, cN and cM categories.

Regarding TNM clinical staging, the cT3 category was the most common, occurring in 40.6% of the cases (Figure 3). Lymph node involvement (cN+) was described in 48.9%, and distant metastases (cM+) in 14.4% of the staging exams. The most common TNM final clinical stage was III (29.4%), followed by II (26%), I (24.2%), and IV (20.5%).

Regarding the intention of the surgical treatment, most patients (74.4%) underwent surgery with curative intent (Table 3). The open access route was predominant (82.8%), while minimally invasive surgery, which includes laparoscopic and robotic access, was performed in 17.2% of the cases. Among the types of surgical procedures, the most common were subtotal gastrectomy (45.3%), followed by total gastrectomy (33.1%), and diagnostic laparoscopy (10.7%). Preoperative chemotherapy was performed in 37.2% of cases, and conversion chemotherapy, in 3.7%.

**Table 3:** Treatment characteristics of all patients included.

Variables	n	%
Intention of surgery		
Curative	467	74.4
Diagnostic	65	10.4
Palliative/Cytoreduction	96	15.3
Preoperative chemotherapy		
No	391	61.6
Yes	222	37.2
Yes - conversion	22	3.7
Surgical access		
Open	453	82.8
Minimally invasive	94	17.2
Type of procedure		
Wedge resection	2	0.3
Diagnostic laparoscopy	67	10.7
Gastrojejunostomy	29	4.6
Gastrostomy	6	1.0
Jejunostomy	14	2.2
Gastric partitioning	6	1.0
Completion total gastrectomy	11	1.8
Subtotal gastrectomy	283	45.3
Total gastrectomy	207	33.1

## DISCUSSION

During the planning of the study, defining the strategy to include centers representative of the population from all regions of the country was a challenge. Fortunately, it was possible to include at least one center from each of the five Brazilian regions. As expected, the cases were predominantly from the Southeast region (40.6%), since it has 41.7% of the Brazilian population, which was approximately 203,062,512 in 2022^8^. The least represented region in the study, with only 3.5% of the cases, was the Midwest, which represents 8.05% of the Brazilian population. Defining the number of participating centers for each region without previously knowing their capacity for inclusion could lead to a great imbalance in regional representation. In the end, we consider that the distribution of cases covered by each region was adequate.

According to a survey by the Brazilian National Supplementary Health Agency, in 2023 more than 51 million inhabitants had some health insurance, a number that represents about 25% of the Brazilian population^9^. Unfortunately, in the present study, less than 10% of cases had some health insurance. This low representation was already expected, as we included only two centers with public and private care (one in the Southeast and one in the South). In Brazilian private supplementary health, there is a greater distribution of neoplasm cases among private General Hospitals, with less centralization of cases in Cancer Centers. Therefore, there was difficulty in including more private centers with multidisciplinary teams dedicated to cancer treatment, high volume of cases, and conducting research related to GC, as found in the public centers participating in the study^6^.

As expected, we found a predominance of male patients in the sixth decade of life^10^ ^,^ ^11^. What caught our attention was the good clinical condition, as evaluated by the ECOG and ASA classification. This may be related to the inclusion of only patients with GC who underwent a surgical procedure. Therefore, patients with advanced metastatic disease who tend to display worse performance were not included^12^.

During clinical staging, a quarter of the patients underwent diagnostic laparoscopy. Considering that approximately 25% of the cases had clinical stage I, in which diagnostic laparoscopy is not necessary, the proportion of patients who were referred for the procedure becomes even higher. We were pleased to see the widespread use of diagnostic laparoscopy in the country. Diagnostic laparoscopy has already been established as an important staging modality for the detection of unsuspected peritoneal metastases and can alter management in up to 30% of cases^13^ ^,^ ^14^. Performing it before the preoperative chemotherapy also allows one to define whether the treatment will be neoadjuvant, conversion therapy, or just palliative. In centers that have a research protocol using intraperitoneal CTX modalities, laparoscopy also allows the recruitment of patients with peritoneal metastases^15^ ^-^ ^17^.

On the other hand, the use of EUS and PET scans was rare. Currently, the main indication for EUS is to define the depth of tumor invasion in cases considered for endoscopic resection^18^. Once again, the inclusion criteria of the study based only on patients undergoing surgical procedures meant that cases undergoing endoscopic resection were not included, which would probably contribute to a higher number of EUS examinations. Another indication of the EUS included the evaluation of lymph node involvement in patients who are potential candidates for preoperative chemotherapy. The low frequency of EUS use demonstrates a preference for computed tomography for lymph node staging in our country. PET scan has been increasingly used in the staging of neoplasms, but its accuracy in GC is not high, with better performance in proximal tumors and intestinal histological type^14^. The low frequency of PET scans in the present study is in line with this concept and with the fact that more than 90% of the institutions are public, with greater difficulty in accessing the exam, which is expensive, and may have influenced results.

Regarding the histological type, the diffuse Lauren type was predominant over the intestinal one. Previously, there was a predominance of the intestinal type in the world, but the reduction in the occurrence of known risk factors such as smoking, H. pylori infection, and consumption of processed foods led to a change in this proportion^19^ ^-^ ^22^. Previously published national case series described a preponderance of the intestinal type, while others described a predominance of the diffuse type^10^ ^,^ ^21^ ^,^ ^22^. Again, regional and temporal differences may be responsible for these conflicting results, but our findings suggest the predominance of the diffuse type in Brazil in operated patients.

The most frequent clinical stage was cTNM III, but almost a quarter of the patients were classified as stage I. This high proportion caught our attention, but since we do not have data available on all GC cases in the population, it is not possible to affirm that there was an increase in early diagnosis. This increase in stage I may be due to the better staging of metastatic tumors that were previously considered as stage II/III. With adequate staging, these metastatic patients were referred to exclusive palliative chemotherapy and were not included in the study, reducing the proportion of stages II/III among the operated cases.

The indication for curative resection was the most common in the included cases, with subtotal distal gastrectomy being the most common procedure. Almost 20% of the patients underwent the minimally invasive approach, demonstrating an increase in its indication^22^ ^-^ ^25^. Given the high frequency of stage I and distal clinical tumors, which are the most frequently selected cases for minimally invasive approaches, an increase in the indication of this route is expected in the future in Brazil^26^. Finally, almost 40% of patients underwent preoperative CTX, demonstrating alignment with the worldwide trend of indicating CTX before the surgical procedure whenever possible and tolerated^4^.

The present study has some limitations. Only reference institutions were included in the treatment of GC. Thus, the reality of treatment in non-specialized centers was not represented. It has been previously reported that patients operated on in non-specialized centers have worse surgical and oncological outcomes. Currently, Brazil has more than 300 health establishments authorized by the Ministry of Health to treat cancer patients divided between High Complexity Care Units (UNACON), High Complexity Oncology Care Centers (CACON), and General Hospitals with oncological surgery. Although significant, this number is still insufficient to care for all cancer patients, and many still undergo surgery outside the oncology care network. As previously mentioned, the inclusion of patients undergoing surgical procedures excluded cases with metastatic disease and initial tumors undergoing endoscopic treatment. Another limitation was the lack of complete data on all the variables of the patients included. To overcome this limitation, the percentages were always presented to the total number of cases available for each variable, and not to the total number of patients included in the study.

As strengths, we highlight that this is the largest multicenter study related to GC conducted in our country, which included referral centers from all geographic regions. The panorama for GC treatment found was consistent with those reported in the literature, with the most unprecedented results related to the increase in referrals for preoperative chemotherapy and expanded access to minimally invasive surgery, consistent with global trends. Finally, the present study meets the objectives of the ABCG to disseminate and improve GC treatment in Brazil. Future analyses of data related to long-term survival of the cohort will be presented.

## CONCLUSION

The present study allowed us to evaluate the current panorama of surgical treatment of Gastric Cancer, representing all regions of Brazil. Although stage III continues to predominate, there was also a relevant frequency of stage I clinical tumors, as well as a trend towards the use of diagnostic laparoscopy in the staging of the disease. We found a higher proportion of distal and diffuse tumors, with a predominance of distal curative resections. Finally, the use of preoperative chemotherapy and minimally invasive surgery has become increasingly common in the country.siva tem se tornado cada vez mais comum no país.
